# Phytoconstituents-mediated Targeting of Ferroptosis for the Treatment of Cardiovascular Disease

**DOI:** 10.2174/011573403X370981250618074406

**Published:** 2025-06-24

**Authors:** Parul Gupta, Anjali Sharma, Sachin, Shubham Sharma, Devkant Sharma

**Affiliations:** 1 Department of Pharmaceutics, Guru Gobind Singh College of Pharmacy, Yamunanagar (135001), Haryana, India;; 2 Department of Pharmaceutics, Ch. Devi Lal College of Pharmacy, Jagadhri (135003), Haryana, India

**Keywords:** Ferroptosis, CVD, phytoconstituents, redox balance, atherosclerosis, resveratrol, quercetin

## Abstract

Ferroptosis, an instance of iron-dependent programmable cell death that results from oxidative stress & lipid peroxidation, has garnered interest due to its associations with cardiovascular diseases, such as atherosclerosis, myocardial infarction, as well as heart failure. Unlike necrosis or apoptosis, ferroptosis involves unique metabolic pathways that disrupt cellular redox balance and lipid homeostasis, leading to substantial cell damage in cardiovascular tissues. It is becoming recognized that phytoconstituents—bioactive compounds derived from plants—can modify ferroptosis pathways and provide cardioprotective advantages. Compounds including curcumin, resveratrol, quercetin, tanshinone IIA, and epigallocatechin gallate (EGCG) have shown potential in preclinical studies by concentrating on significant ferroptotic processes. Finally, by controlling iron homeostasis, boosting antioxidant responses (such as Nrf2 pathway activation), and reducing lipid peroxidation, these phytochemicals may mitigate ferroptosis-induced cardiac cell death. In animal studies, these natural compounds have shown promise in reducing oxidative damage and improving heart function after injury. This article summarises the mechanisms *via* which a variety of phytoconstituents influence ferroptosis and discusses their potential as an adjuvant treatment for CVD. While these findings are encouraging, further research is needed to use them in clinical settings, with a focus on long-term safety in human populations, optimal dose, and absorption. The cardioprotective properties of phytoconstituents, which focus on ferroptosis, may provide a unique, plant-based therapeutic strategy for the treatment of CVDs.

## 1 INTRODUCTION

Cardiovascular disease (CVD) is a major cause of mortality worldwide [1]. According to the World Economic Forum, more than 50% of fatalities from noncommunicable diseases are attributable to cardiovascular disease (CVD), which is expected to claim the lives of over twenty-two million people by 2030. According to the American Heart Association, CVD will cost about 1.1 trillion dollars by 2035 [2]. Understanding the pathophysiology of CVD, as well as identifying its treatment targets, is crucial since it has grown to be a significant public health problem due to the high death rate as well as high healthcare costs. Cell death is the ultimate result of cardiomyocyte damage brought on by CVD, regardless of the exact mechanism.

Numerous essential physiological processes, including development, immunity, and tissue dynamic homeostasis, are predicated on cell death. Scientists have traditionally distinguished between 2 types of cell death: the first one is necrosis and the second is apoptosis. Numerous recent investigations have shown that cell death, which was formerly thought to be unintentional, is tightly controlled. As a result, ferroptosis has gained significant attention in recent research. Ferroptosis, first proposed by Dixon in 2012 [3], is characterized by intracellular iron accumulation, elevated lipid peroxidation, mitochondrial shrinkage, reduced cristae, increased mitochondrial membrane density, accumulation of iron-associated free radicals, and depletion of glutathione (GSH).

Ferroptosis is different from apoptosis and autophagy. Iron-chelating drugs and lipophilic antioxidants may be able to stop ferroptosis, a new kind of PCD, but they are unable to block autophagy inhibitors, necrosis, or conventional apoptosis. According to recent research, ferroptosis has a major impact on CVD. Nevertheless, it is still unknown what molecular processes underlie ferroptosis's impact on CVD and how it interacts with other forms of PCD.

Scientists have been interested in herbal medications because of their exceptional safety and effectiveness. A growing body of research suggests that certain herbal remedies may effectively treat cardiovascular disease due to their anti-inflammatory, anti-apoptotic, and antioxidant properties. The significance of iron homeostasis in CVD has garnered a lot of attention lately, particularly in relation to iron overload-induced cardiomyocyte ferroptosis.

Natural ingredients (phenylpropanoids, flavonoids, terpenoids, and polyphenols) and Chinese herbal compounds (injection of Salvia miltiorrhiza, Gegen Qinlian decoction, Tongxinluo, and Banxia-Houpu decoction) have been found to regulate iron overload, specifically through ferroptosis-related pathways and targets, in the search for potential CVD drugs. Tongxinluo and Salvia miltiorrhiza are traditional folk remedies that are often used in China to treat cardiovascular disease. The traditional medicinal use of Salvia miltiorrhiza Bunge to 'promote blood circulation and remove blood stasis' may help reduce inflammation and protect organs from damage caused by iron overload. *Astragalus membranaceus* (Fisch.) Bunge, *Gardenia jasminoides* J. Ellis, and *Chuanxiong Rhizoma* are well-known cardioprotective herbs that have been shown to lower iron overload and promote heart health. Among the active ingredients in these plants are geniposide, astragaloside IV, and tetramethylpyrazine. Numerous flavonoids have shown potent iron-chelating properties against iron deposition, including those used in traditional Chinese remedies for cardiovascular protection [3]. Phytoconstituents' potential as ferroptosis-targeting medications in the treatment of CVD is further enhanced by their affordability and accessibility. In contrast to synthetic medications, which may need substantial research, development, and regulatory approval, many phytoconstituents are easily accessible as dietary supplements or ingredients in functional foods. Their broad availability and cheaper manufacturing costs make them a desirable alternative, especially in low-resource environments where access to traditional CVD medications may be restricted. Furthermore, since they are found naturally and often have fewer adverse effects than synthetic ferroptosis inhibitors, phytoconstituents are better suited for long-term usage in the treatment of CVD.

A comprehensive literature review was conducted using electronic databases such as PubMed, Scopus, and Google Scholar. The selected literature was evaluated based on its relevance, study quality, and methodological robustness. This methodological framework ensures the review process's transparency, reproducibility, and scientific rigor. The purpose of this study is to discuss iron metabolism in cardiomyocytes, with a focus on ferroptosis. A thorough analysis of herbal remedies that could guard against iron overload in cardiovascular disease is also included in this research.

## 2 FERROPTOSIS

The Fenton reaction, a chemical interaction between hydrogen peroxide and ferrous iron, produces an active component that may oxidize intracellular molecules [4]. It is currently thought that lipid hydroperoxide buildup, which lowers the limit of glutathione peroxidases (GPXs), is the cause of ferroptosis. Lipid peroxides and free iron ions in cells then combine to form lipid free radicals *via* the Fenton reaction, which leads to dying cells at the end. Thus, lipid peroxidation, GSH production and consumption, and aberrant iron metabolism are the three primary processes of ferroptosis, as shown in Fig. (**1**).

### 2.1 Peroxidation of Lipids

Ferroptosis is mostly caused by membrane phospholipids, which are found in copious quantities in polyunsaturated fatty acid (PUFA)-phosphatidylethanolamines (PEs) [4]. The main components of PUFA-PEs, which are made from PUFAs present in the cell membrane, are arachidonic acid (AA) and adrenic acid. Polyunsaturated fatty acyl-coenzyme A (PUFA-CoA) is created when PUFAs are acylated by acyl-CoA synthetase long-chain family member 4 (ACSL4). Lysophosphatidylcholine acyltransferase 3 (LPCAT3) esterifies PUFA-CoA, which then joins PE to create PUFA-PEs [5]. Lipid peroxide, the primary product of lipid peroxidation, is produced when PUFA-PEs are oxidised by lipoxygenase (LOX)-mediated enzymatic activities on the plasma membrane and endoplasmic reticulum. These reactions may produce secondary chemicals, such as molecular aldehydes and polymer aldehydes [6]. Several research has demonstrated the significance of LOX and ACSL4 in controlling lipid peroxidation in this process. One research, for instance, discovered that the ACSL4 gene is highly expressed in breast cancer cells and that its knockdown lowers the synthesis of PUFA-PEs (ferroptosis inhibitors), which prevents ferroptosis caused by RSL3 [7]. Inhibition of 5-LOX and 15-LOX may further decrease RSL3-induced cell death [8], even while pharmacologic inhibition and deletion of the ACSL4 gene improve resistance to RSL3-induced ferroptosis [9].

### 2.2 GSH Synthesis and Utilisation

To protect cells from oxidative damage, a dynamic balance between the synthesis and consumption of glutathione (GSH) is essential. GSH is primarily synthesized through enzyme-catalyzed reactions involving intracellular L-cysteine (L-Cys), glutamate (Glu), and glycine (Gly) [10], with L-Cys being the most critical rate-limiting precursor. Cellular L-Cys levels largely depend on the uptake of extracellular cystine (Cys-Cys) *via* the glutamate/cystine antiporter system x(c)(−), which consists of SLC7A11 (solute carrier family 7 member 11) and SLC3A2 (solute carrier family 3 member 2), a transporter with a 12-transmembrane domain [11].

When cystine uptake is impaired, an alternative route—the trans-sulfuration pathway—can compensate by producing L-Cys endogenously. This process is catalyzed by cystathionine-β-synthase (CBS) and cystathionine-γ-lyase (CGL) [12]. Additionally, glutamine uptake from the extracellular environment, mediated by SLC38A1 and SLC1A5, plays a role in maintaining Glu levels. Once inside the cell, glutaminase 2 (GLS2) converts glutamine into Glu, thereby supplying a key substrate for GSH synthesis [13].

Another important regulatory pathway involves Ferroptosis Suppressor Protein 1 (FSP1), coenzyme Q10 (CoQ10), and nicotinamide adenine dinucleotide phosphate (NADPH). NADPH is a crucial electron donor involved in redox balance and anabolic processes across all living systems. It not only supports GSH synthesis indirectly but also plays a key role in regenerating GSH from its oxidized form, glutathione disulfide (GSSG), *via* the enzyme glutathione reductase, a reaction that also produces NADP(+) [14].

Once synthesized, GSH acts as a co-substrate for glutathione peroxidases (GPXs), particularly GPX4, which neutralize reactive oxygen species (ROS) by reducing lipid peroxides [15]. However, components of the NADPH oxidase (NOX) protein family use NADPH to convert oxygen into superoxide, a type of ROS, thereby promoting ferroptosis [16]. These findings suggest that NADPH has dual roles in ferroptosis-supporting antioxidant defenses while also potentially contributing to ROS generation. The precise role of NADPH homeostasis in the regulation of ferroptosis remains an area requiring further investigation [17-20].

### 2.3 Improper Metabolism of Iron

Many essential metabolic processes depend on iron, a protein cofactor. For instance, transferrin (TF) and iron in the blood mix to form the TF-Fe^3+^ combination [21]. It is possible for the circulating TF-Fe^3+^ complex to attach to the cell membrane's TF receptor 1 (TFR1) protein and be taken up by the cells and placed in endosomes. Prostate's six-transmembrane epithelial antigen 3 (STEAP3) may then convert Fe^3+^ in the endosomes to Fe^2+^. Fe^2+^ may enter the cytoplasm *via* Zrt-Irt-like proteins (ZIPs) or the divalent metal-ion transporter-1 (DMT1). A free iron pool is then formed in the cytoplasm by the remaining Fe^2+^, while a portion of the Fe^2+^ in the cytoplasm attaches to ferritin heavy chain 1 (FTH1) and is oxidised to Fe^3+^, which subsequently joins to ferritin light chain (FTL) to create a ferritin complex that is stored in the cell [22]. Poly(rC) binding proteins (PCBPs) interact with Fe^2+^ in the free iron pool. For instance, ferritin, nonheme ferritin, and other molecules are formed when Fe^2+^ attaches to PCBP1 and protein with PCBP2's help [23]. To maintain GSH's stability, Fe^2+^ may attach to its L-Cys residue. The transmembrane protein ferroportin 1 (FPN1) may carry the excess Fe^2+^ out of the cell and proceed with blood transport [24]. Ferritin and TFR1's functions in ferroptosis are being investigated. For instance, Yang *et al*. discovered that erastin-induced ferroptosis might be lessened by downregulating TFR1 expression [25]. Moreover, heat shock protein B1 (HSPB1) reduces intracellular iron levels and increases cell resistance to ferroptosis in this manner [26]. On the other hand, by upregulating TFR1 expression, iron regulatory protein (IRP) and hypoxia-inducible factor 1 (HIF-1) may enhance iron absorption and decrease cell resistance to ferroptosis [27]. FTH1, FTL, and Fe3+ work together to create ferritin, a stored active form of antioxidant iron that may prevent iron-mediated lipid peroxidation and is another regulator of ferroptosis [28]. Some investigations have discovered that iron response element binding protein 2 (IREB2) reduces the vulnerability of cells to ferroptosis by blocking the creation of FTL and FTH1 [29]. According to another study, autophagy triggers ferritinophagy, which is when Ferritin is transported to lysosomes for breakdown by nuclear receptor coactivator 4 (NCOA4). This encourages ferroptosis and raises the quantity of free intracellular iron. On the other hand, ferroptosis is encouraged and ferritin levels are raised by NCOA4 overexpression [30]. These findings imply that autophagy activation controls intracellular iron homeostasis, which in turn triggers ferritinophagy and encourages ferroptosis. Increased TF saturation and development of non-TF-bound iron (NTBI) are other signs of iron overload. Oxidative stress and tissue iron loading in the bloodstream are direct causes of NTBI, a potentially harmful type of iron [31]. High TF saturation is the most common cause of NTBI. The kinetic balance between iron excretion into serum, binding by TF, removal from circulation, and utilisation in circulation is shown by the occurrence of NTBI, which cannot be regarded as a simple phenomenon of TF supersaturation [32]. Non-transferrin-bound iron (NTBI) has been reported in the blood of diabetic patients with partially saturated transferrin (TF) levels-*i.e*., less than 50% TF saturation—as well as in some healthy individuals [33-35].

Numerous tissues and organs suffer oxidative damage as a result of NTBI's elevated redox activity [36]. The buildup of NTBI may harm organ cells, especially those in the heart. Furthermore, intracellular Fe^2+^ transport may also be mediated by specific calcium channels. For instance, L-type calcium channel (LTCC) blockers dramatically decreased the absorption of NTBI by cardiomyocytes [37]. Conversely, T-type calcium channels (TTCC) have a major impact on the mechanism of iron absorption by thalassemic cardiomyocytes [38]. By using the appropriate inhibitor channel inhibitors, myocardial iron deposition was considerably reduced, and heart function was enhanced. Recent research, however, has shown that TTCC does not stop Fe^3+^ absorption into cardiomyocytes when it is administered to cultured thalassaemia cardiomyocytes after LTCC. This suggests that a different mechanism may be involved in the absorption of Fe^3+^ in Mediterranean cardiomyocytes [39]. With the aid of catecholamines, lipocalin 2 (Lcn2) firmly binds to Fe^3+^, blocking its conversion to Fe^2+^. According to earlier research, elevated Lcn2 synthesis raises adipose tissue iron levels, which starts the negative consequences of iron overload [40]. According to recent research, cardiomyocytes experiencing Fe^3+^ overload benefit from suppressing Lcn2 because it lowers Fe^3+^ uptake [41].

## 3 FERROPTOSIS IN CARDIOVASCULAR DISEASE

Additionally, one of the causes of CVD is the loss of distinct cardiac muscle cells. The end outcome of CVD, which often starts with vascular anomalies, is heart failure (HF). Although ferroptosis, another kind of cell death, is also significant in CVD, it has long been thought that L-Cys protease-dependent apoptosis is the primary mechanism of cardiomyocyte death. Aneurysm, arterial coarctation, heart valve disease, cardiac arrhythmia, cardiomyopathy, atherosclerosis, myocardial infarction (MI), myocardial ischemia-reperfusion (I/R), and heart damage are all associated with ferroptosis [41]. In Fig. (**2**), the precise mechanism is shown. Some CVDs may benefit from ferroptosis as a therapeutic target, according to research. Cyanthidin-3-glucoside, resveratrol, liproxstatin-1, dexmedetomidine (Dex), ferrostatin-1 (Fer-1), dexrazoxane (DXZ), xanthohumol, baicalin, and naringenin are some possible therapies for I/R. Reducing ROS production and preserving GPX4 activity are the two primary molecular pathways for I/R. The reduction of reactive oxygen species (ROS) generation is a key molecular mechanism in the prevention and treatment of cardiomyopathy. Compounds such as astragaloside IV (AsIV), DXZ, ferrostatin-1 (Fer-1), and MitoTEMPO have shown potential as therapeutic agents. By lowering oxidative stress and preserving GPX4 function, dexamethasone (Dex), astragaloside IV (AsIV), puerarin, and resveratrol may be useful therapeutic agents for myocarditis. Table **1** shows some herbals that target ferroptosis for the treatment of cardiovascular disease.

This figure illustrates how ferroptosis contributes to various cardiovascular conditions, including atherosclerosis, myocardial infarction, cardiomyopathy, heart failure, valve calcification, arrhythmia, and aortic aneurysm. Key mechanisms include oxidative stress, glutathione (GSH) imbalance, iron overload, impaired GPX4 and SLC7A11 function, and activation of the PERK-eIF2α-ATF4-CHOP pathway.

### 3.1 Ferroptosis and Atherosclerosis

Wang *et al*. found that administering Tongxinluo (TXL), a multifunctional Chinese drug, to apoE-/-mice fed a high-fat diet decreased the area of atherosclerotic plaque, improved dyslipidemia, and diminished the disruption of the pulmonary microvascular endothelium barrier. In human pulmonary microvascular endothelial cells (PMECs), TXL enhanced GSH activity and markedly increased the expression of GPX4 and FSP1. Nitric oxide (NO), MDA, and ACSL4 levels decreased concurrently, indicating that TXL delays the progression of AS by inhibiting PMEC ferroptosis [42]. In apoE-/-mice fed a high-fat diet, it has been shown that icariin, the main active ingredient in the traditional Chinese medicine Epimedium grandiflorum Maxim, reduces the size of the atherosclerotic area, controls inflammation, improves dyslipidaemia, and inhibits ferroptosis in aortic tissue. GPX4 and FTH1 expression rose, ROS and Fe^2+^ levels dropped, and autophagy significantly enhanced when icariin was co-incubated with ox-LDL-treated HUVECs. These results suggest that by inhibiting VEC ferroptosis, icariin may be able to improve AS [43]. Additionally, Rong *et al*. found that in diabetic atherosclerotic rats, intraperitoneal injection of hydroxysafflor yellow A (HSYA), an active ingredient of *Carthamus tinctorius* L., dramatically reduced plasma lipid levels, plaque lipid production, and aortic ferroptosis. Incubating HUVECs with HSYA also effectively reversed the ferroptosis caused by high glucose and ox-LDL, as seen by increased cell viability and expression of xCT, GPX4, and GSH and decreased levels of iron, ROS, MDA, and ACSL4. Another study found that HSYA protects HUVECs from ferroptosis by blocking VEC ferroptosis *via* the miR-429/xCT pathway, hence providing atheroprotective advantages [44].

Fluvastatin is a powerful medication that lowers cholesterol and has many anti-atherogenic effects [45]. In ox-LDL-treated HUVECs, fluvastatin dose-dependently enhanced cell migration and proliferation, up-regulated xCT and GPX4 expression and reduced 4-HNE, TNF-α, and IL-1α levels [46]. This implies that by preventing ferroptosis, fluvastatin restores endothelial dysfunction and inflammation brought on by ox LDL.

### 3.2 Ferroptosis and Cardiomyopathy

Ferroptosis is a major contributing component to the cardiomyopathy caused by DOX, according to Fang *et al*. Mice given DOX had iron accumulation in the mitochondria and lipid peroxidation of the membranes. Ferrostatin-1 (Fer-1), an inhibitor of ferroptosis, and dexrazoxane (DXZ), an iron chelator, both significantly reduced DOX-induced cardiac damage and mortality, while inhibitors of apoptosis, necroptosis, and autophagy did not improve the mice's condition. Fer-1 and DXZ protect against DOX-induced cardiomyopathy because they maintain mitochondrial function. Both Fer-1 and DXZ prevented the growth and deformation of the cardiac mitochondria in rats administered DOX. By preventing ferroptosis, the chemicals reduced the size of the infarct and serum markers of heart injury after ischemia/reperfusion. Additionally, Fang *et al*. found that the mitochondria-targeting antioxidant MitoTEMPO prevented ferroptosis by reducing lipid peroxidation, which may eventually reduce DOX-induced cardiomyopathy. Additionally, RNA sequencing revealed that DOX-induced ferroptosis resulted in a substantial up-regulation of heme oxygenase-1 (Hmox1) in murine cardiac tissues. Hmox1 expression rises when nuclear factor erythroid 2-related factor 2 (Nrf2), a redox-sensitive transcription factor, is activated. DOX induces heme to break down and release iron *via* increasing the Nrf2/Hmox1 pathway in the heart. Thus, suppression of ferroptosis may provide protection against cardiomyopathy [47].

### 3.3 Ferroptosis and Myocardial Infarction

Park *et al.* have shown that ferroptotic death of cardiomyocytes occurs during MI. Proteomic analysis of mouse heart tissues after MI by left anterior descending ligation showed that the ferroptosis inhibitor GPX4 protein levels were significantly downregulated throughout the early and middle stages of MI. It was shown that suppression of GPX4 increased the susceptibility of primary neonatal rat ventricular myocytes to ferroptosis when cysteine deprivation reduced GSH levels. MI has been associated with the epithelial-mesenchymal transition (EMT) and ferroptotic-related Nrf2 [48]. By protecting the cells from damaging ROS stress, the master transcription factor Nrf2 regulates antioxidant responses and stops ferroptosis in several cell types [49, 50]. ZEB1-mediated lipogenic reprogramming may link ferroptosis susceptibility to EMT [51]. It has been shown that the Nrf2/Hmox1 pathway raises Hmox1 activity during the early and middle stages of MI, causing excess iron that leads to heart cell ferroptosis. EMT signalling is activated when heart cells experience MI, which fosters an environment that is favorable for ferroptosis. Furthermore, Bulluck *et al*. demonstrated that reducing negative left ventricular remodeling in patients with reperfused MI may be achieved by focusing on residual myocardial iron as a therapeutic target [52].

### 3.4 Ferroptosis and Heart Failure

Chen *et al*. suggest that inhibiting ferroptosis and autophagy may reduce cardiac cell death during HF. An integrated bioinformatics analysis revealed that rat heart tissue following heart failure exhibits upregulated differentially expressed genes, including Toll-like receptor 4 (TLR4) and NADPH oxidase 4 (NOX4). Moreover, intramyocardial injections of TLR4-siRNA or NOX4-siRNA lentiviruses reduced the symptoms of heart failure by preventing ferroptosis and autophagy in cardiac cells, hence blocking their corresponding receptors [53]. Despite its therapeutic use, the exact mechanisms by which puerarin improves heart failure remain unclear. Liu *et al*. found that puerarin restored cell viability and prevented ferroptotic death of myocytes brought on by either erastin or isoprenaline. Purerarin treatment reduced NOX4 expression while increasing GPX4 expression. Puerarin treatment improved striated muscle organisation, prevented ferroptosis, and decreased mitochondrial atrophy in rats with pressure-overload-induced HF [54].

#### 3.4.1 Natural Remedies that Prevent Doxorubicin (DOX)-induced Ferroptosis in the Heart

Natural remedies have long been used to cure human illnesses. Their low toxicity and well-established safety profile have been widely acknowledged. The problem of DIC has also been thoroughly studied in relation to natural products. Scientists are starting to evaluate the part natural products play in DOX-induced ferroptosis as fresh ideas and more studies into the mechanism of DIC have surfaced. To offer new insights and recommendations for future research on the supportive role of traditional Chinese medicine (TCM) in anti-tumor therapy, this section examines natural compounds that modulate doxorubicin (DOX)-induced myocardial ferroptosis.

## 4 RESVERATROL

One well-known polyphenolic substance that is a member of the stilbene derivative category is resveratrol. This naturally occurring phytoalexin is mostly found in red wine, peanuts, rhubarb, mulberries, and grapes. Potential protective benefits against cardiovascular disorders have been suggested for resveratrol [72, 73]. Numerous studies have also been conducted on resveratrol's ability to prevent DIC. The groups treated with DOX alone did not benefit as much as those treated with resveratrol and DOX together, based on the results of studies conducted both *in vitro* as well as *in vivo* [74]. Heart weight, cell viability, cell death, and biological and histological changes in the heart's cells and tissue were the main factors impacted by these factors. Resveratrol's cardioprotective effects are mostly brought about by its antioxidant, anti-inflammatory, and anti-apoptotic qualities [75]. Additionally, resveratrol has shown potential as a therapeutic method for DIC alleviation by altering ferroptosis and acting as a potent p62 activator. Its shielding action was comparable to that of ferrostatin-1, an extensively studied inhibitor of ferroptosis. The safeguarding properties of resveratrol were believed to be due to the activation of the p62-NRF2 axis [76]. Chen *et al*. used neonatal rat cardiomyocytes, H9c2 cardiomyoblasts, as well as C57BL/6J mice to produce DIC models. They discovered that resveratrol successfully inhibited ferroptosis, most likely by controlling the signalling pathway of mitogen-activated protein kinase (MAPK) [77].

### 4.1 Gallate of Epigallocatechin

Green tea's most prevalent catechin, epigallocatechin-3-gallate, accounts for more than half of its overall content of polyphenols and active ingredients [78]. The structure's strong antioxidant and free radical scavenging capabilities are attributed to several phenolic hydroxyl pairs. In addition to killing drug-resistant cancer cells, epigallocatechin-3-gallate may increase the efficacious outcome of chemotherapeutic medications, including paclitaxel, tamoxifen, DOX, as well as cisplatin. Along with reducing the negative effects of chemotherapy, such as ototoxicity, gastrointestinal problems, and cardiotoxicity, it also lessens the development and incidence of tumor-induced cachexia [79]. Cardiomyocytes may be efficiently protected against DIC by epigallocatechin-3-gallate, which blocks oxidative stress and inflammation as well as apoptotic signaling pathways [80-83]. By lowering iron buildup, preventing oxidative stress, and restoring normal lipid metabolism, pretreatment with epigallocatechin-3-gallate has been shown to lessen Ferroptosis brought on by DOX in DIC models both *in vitro* as well as *in vivo*. These advantages were brought about by the overexpression and phosphorylation of AMPKα2, which conserved mitochondrial function, improved energy availability, and encouraged adaptive autophagy [84].

### 4.2 Baicalin

The medicinal herb baicalin, or Scutellaria baicalensis Georgi, is a member of the Lamiaceae family. Because of its multiple uses, it has a long history and is highly valued in Chinese herbal medicine. Flavonoids, which include scutellarein, wogonin, and wogonoside, the primary biologically active parts of S. baicalensis, are baicalin and baicalein [85]. Flavonoids such as scutellarein, wogonin, and wogonoside are among the main biologically active components of *Scutellaria baicalensis*; however, baicalin and baicalein are considered its primary active constituents [86]. A clear cardioprotective action against DIC was shown by baicalin, which blocked its own natural inhibitor Dickkopf-1 (DKK1). Consequently, the toll-like receptor 4/nuclear factor-kB (TLR4/NF-κB) transmission sequence was disturbed, and the canonical Wnt/β-catenin cascade was enhanced [87]. Additionally, Feng *et al*. [88] obtained related results. Additionally, Zeng *et al*. successfully produced baicalin-peptide supramolecular-formed nanofibers that are specifically designed to interfere with the angiotensin II type 1 receptor (AT1R). By efficiently scavenging cardiac superoxide and inhibiting cardiomyocyte ferroptosis, the exceptional targeting capacity towards damaged cardiac muscle cells led to a significant therapeutic impact on DOX-induced myocardial injury [89, 90].

### 4.3 Cinnamon Aldehyde

An aromatic aldehyde molecule, cinnamon aldehyde, is used in both industrial and culinary applications as a natural flavoring and fragrance element. It is the primary ingredient in cinnamon and makes up around 98% of the bark's essential oil. A thick, amber, or golden liquid with an oddly pleasing scent is how cinnamon essential oil is characterized [91]. Crucially, a transformation is assumed by all-natural cinnamon aldehydes [92]. Transient receptor potential ankyrin 1 (TRPA1) deficiency increased mortality, cardiac dysfunction, and left ventricular remodelling in rats with DOX-induced dilated cardiomyopathy. Cinnamon aldehyde, a well-known TRPA1 activator, decreased the synthesis of the inflammatory protein S100A8 and enhanced cardiac function [93]. According to a research by Mao *et al*., cinnamon aldehyde had comparable cardioprotective effects in rats and DOX-treated H9c2 cells. Mechanistically, cinnamon aldehyde reduced DOX-induced cardiomyocyte ferroptosis by significantly upregulating HO-1 expression and encouraging the nuclear translocation of Nrf2. However, the positive effects of cinnamon aldehyde on increasing cardiomyocyte lifespan were negated by the addition of erastin, a ferroptosis agonist [94].

### 4.4 Capsaicin

The alkaloid component capsaicin exists in most plants of the genus Capsicum, especially red peppers and chillies. Due to the presence of capsaicin, which gives them their distinct texture and hot flavour, these plants are often employed as culinary ingredients. In addition to its culinary uses, capsaicin has drawn interest for its medicinal uses, which include treating psoriasis, allergic rhinitis, inflammatory and neuropathic pain, and itching [95]. Additionally, it may be used to treat metabolic problems, cardiovascular illnesses, and tumours. Its effect on cellular iron metabolism and lowering iron buildup has been shown in recent research [96]. Capsaicin reduced cardiac ferroptosis and enhanced cellular iron homeostasis in DOX-treated C57BL/6 J mice as well as H9c2 cells by enhancing solute carrier 40-member 1 (SLC40A1) and decreasing transferrin (Trf) expression. Furthermore, *via* altering the PI3K-Akt signalling pathway, capsaicin seemed to reduce DOX-induced apoptosis [97].

### 4.5 Fisetin

One naturally occurring polyphenolic flavonoid is fisetin, which may be found in strawberries, apples, cucumbers, onions, persimmons, and other fruits and vegetables [98]. Fisetin contains anti-inflammatory, anti-cancer, anti-angiogenic, and antioxidant properties, among other pharmacological advantages [99, 100]. It is cardioprotective against DIC, according to studies. In research where male SD rats were given DOX, fisetin had cardioprotective benefits by lowering oxidative stress, inflammation, and apoptosis [101]. By blocking insulin-like growth factor II receptor (IGF-IIR) expression through oestrogen receptor-α/-β activation and hsp70-interacting protein/sirtuin 1 (CHIP/SIRT1)-mediated heat shock factor 1 (HSF1) signalling pathways, fisetin lowers DIC in H9c2 cardiomyoblasts and ovariectomised rat models [102]. Li *et al*. also identified a similar protective effect and clarified the ferroptosis mechanism [103]. The results showed that DOX decreased GPX4 and GSH while increasing ROS and malondialdehyde (MDA) levels both *in vivo* and *in vitro*. However, fisetin therapy may reverse these changes. Due to increased SIRT1 and Nrf2 activity, this impact was mediated *via* increased expression of downstream HO-1 and ferritin heavy chain 1 (FTH1) genes. Targeted SIRT1 knockdown diminished the positive effects of fisetin on H9c2 cells.

### 4.6 Licochalcone A

Mostly extracted from liquorice roots, licochalcone A is a bioactive flavonoid with a distinct chalcone structure. Many TCM medications, such as Huoxiang Zhengqi Tincture, compound Glycyrrhiza Oral Solution, and compound Liquorice Tablets, often include liquorice as an adjuvant herb [104]. Researchers have focused on the function of liquorice because of its special therapeutic qualities. The information that is now available indicates that licochalcone A has a wide range of pharmacological characteristics, such as beneficial control of blood sugar and cholesterol levels, as well as anti-inflammatory, antioxidant, and neuroprotective qualities [105]. Licochalcone A at low doses showed antioxidant action against DOX-induced chromosomal damage in Chinese hamster ovary cells, but it had no discernible effect on DOX-induced genotoxicity in mouse erythrocytes [106]. Licochalcone A demonstrated cardioprotective efficacy in a DIC mouse model by improving serum biomarker levels, histological features, and electrocardiographic abnormalities. Additionally, it improved the vitality of DOX-produced H9c2 cells. By raising GSH/GSSG levels and lowering ROS, ferrous iron, and MDA levels, pretreatment with licochalcone A decreased DOX-induced ferroptosis. Licochalcone A may activate the phosphatidylinositol 3-kinase/protein kinase B/phosphorylating murine double minute 2/p53 (PI3K/AKT/MDM2/p53) pathway, according to a mechanistic study [107].

### 4.7 The Taxofolin

Pseudotigusa taxifolia bark is the source of the flavonoid taxifolin, commonly referred to as dihydroquercetin. Numerous therapeutic plants, such as *Larix gmelinii*, *Silybum marianum* L, *Catha edulis*, and *Allium cepa* L, contain it [108]. Among its many pharmacological activities, taxifolin is widely recognized for its anti-inflammatory and antioxidant properties. Numerous studies have reported significant progress in its potential for treating cancer, cardiovascular diseases, acute lung injury, gastric ulcers, Alzheimer's disease, SARS-CoV-2 infection, and other conditions [109]. Additionally, taxifolin increases the efficacy of chemotherapeutic medications while reducing their adverse side effects [110, 111]. Lin *et al*. claim that taxifolin effectively lowers oxidative stress and ferroptosis, minimizing the structural and functional harm that DOX causes to the myocardium. This protective effect was achieved by altering the miR-200a-Keap1-Nrf2 signaling pathway, which raised Nrf2 expression and therefore, upregulated downstream proteins, including HO-1 and NAD(P)H quinone dehydrogenase 1 [112].

### 4.8 Astragaloside IV

The Astragalus membranaceus plant yielded Astragaloside IV, a bioactive triterpene saponin [95]. With xylopyranosyl and glucopyranosyl residues at positions C-3 and C-6, respectively, it is a significant quality control signal for A. membranaceus, according to the Chinese Pharmacopoeia [113]. The cardioprotective drug astragaloside IV is widely used. The study found that Astragaloside IV activated the mitochondrial PI3K/Akt pathway, reducing DOX-induced cardiomyocyte apoptosis without compromising DOX's anti-tumor properties [114]. Lin *et al*. provided further proof that Astragaloside IV lowers oxidative stress brought on by NADPH oxidase 2 and 4 (NOX2 and NOX4), which in turn lowers apoptosis and cardiac fibrosis in DOX-induced cardiomyopathy [115]. Astragaloside IV inhibits NLRP3-mediated pyroptosis and activates the Nrf-2/HO-1 signalling pathway, which Chen *et al*. found protects against DOX-induced cardiac failure [116]. Additionally, astragaloside intravenous treatment decreased DOX-induced cardiac fibrosis and ferroptosis in male SD rats by upregulating GPx4 expression and activating the Nrf2 signaling pathway [117].

### 4.9 Steviol

Steviol, an ent-kaurane diterpenoid, is formed by the hydrolysis of stevioside [118]. The anti-inflammatory, renoprotective, cardiovascular, anti-diabetic, and anti-cancer properties of steviol's pharmacological activity have all been studied. A variety of derivatives for antibacterial, vasorelaxant, cholinesterase, α-glucosidase, antituberculosis, anticancer, and sweetening purposes have been developed because of steviol's structural alterations [119]. Xu and colleagues synthesised 30 steviol derivatives and evaluated their preventive potential using a model of DOX-induced zebrafish cardiomyopathy. According to the results, steviol may have cardioprotective benefits. Further modifications resulted in a number of derivatives with enhanced effectiveness, especially derivatives 16d and 16e, which prevented ferroptosis and shielded H9c2 cells and zebrafish against DOX-induced cardiomyopathy. In addition to halting lipid peroxidation, excessive ROS generation, iron buildup, and GSH depletion, this also restored the loss of mitochondrial membrane potential [120].

### 4.10 Emodin

This natural anthraquinone derivative is found in Emodin, Rheum palmatum, Polygonum multiflorum, Polygonum cuspidatum, and other Chinese medicinal herbs. For more than two millennia, these herbs have been used in traditional medicine, and they are still a key component of many Chinese patent medications [121]. Emodin has been shown to have significant pharmacological and therapeutic benefits, including the ability to cure several cancers, heart conditions, diabetes complications, liver diseases, and asthma [122]. Emodin treatment decreased myocardial damage and DOX-induced cardiac dysfunction in C57BL/6 mice, according to Dai *et al*. To prevent DOX-induced mitochondrial damage and GSDMD-mediated pyroptosis, emodin binds directly to gasdermin D (GSDMD) and prevents its activation [123]. According to a thorough investigation that includes gut microbiota, untargeted metabolomics research, and cardiac function evaluation, emodin may protect DOX-induced myocardial damage and ferroptosis, both of which depend on intact gut microbiota. Research on faecal microbiota transplantation found that emodin repaired the disturbed gut microbiota brought on by DOX and corrected the alterations in blood metabolites. A direct connection between these effects and the vital Nrf2 gene was discovered [68].

### 4.11 Aloe-emodin

Antiviral, antibacterial, anti-inflammatory, neuroprotective, and renal protective qualities are shown by aloe-emodin, a significant bioactive component obtained from R. palmatum and Aloe barbadensis [124, 125]. Wu *et al*. claim that aloe-emodin initiated cellular ferroptosis by selectively inhibiting glutathione S-transferase P1 (GSTP1), a crucial protein involved in ferroptosis. In the meanwhile, they created biomimetic nanocrystals containing aloe-emodin, which successfully induced fast ferroptosis and showed encouraging outcomes for the therapy of cancer [126]. Aloe-emodin, however, was identified by He *et al*. as a new ferroptosis inhibitor that successfully reduced DIC in rat cardiomyocytes. The results showed that aloe-emodin reduced DOX-induced oxidative stress in H9c2 cells while maintaining DOX's anti-tumor effects. Aloe-emodin stopped DOX-induced ferroptosis by restoring downstream signals, SLC7A11 and GPX4, which triggered the Nrf2 pathway. Additionally, aloe-emodin combined with bivalent iron generates a compound that regulates intracellular iron metabolism and reduces lipid peroxidation [127]. The different microorganisms that cause the illnesses may be to blame for these two different results.

### 4.12 Echinacoside

Echinacoside, a naturally occurring phenylethanoid glycoside, is found in the rhizome of Echinacea angustifolia. At around 30%, Cistanche tubulosa contains the highest amount of echinacoside. It is also the main bioactive ingredient in a variety of other botanical sources [128]. Anti-inflammatory, anti-apoptotic, antioxidant, neuroprotective, hepatoprotective, and anti-tumor actions are among the noteworthy pharmacological properties of echinacoside, according to recent pharmacological research [129, 130]. It has significant cardioprotective properties. Echinacoside significantly reduced DOX-induced MDA and lactate dehydrogenase (LDH) levels in rat H9c2 cells while restoring GSH levels. By upregulating GPX4 expression and downregulating prostaglandin-endoperoxide synthase 2 (PTGS2) and lipid ROS levels, it significantly decreased DOX-induced cardiomyocyte ferroptosis. Nevertheless, these protective effects vanished when Gpx4 was inhibited. Similar results were obtained in an *in vivo* model of chronic cardiac failure [90].

### 4.13 Cinnamon

In both industrial and culinary applications, cinnamononaldehyde, an aromatic aldehyde molecule, is used as a natural flavouring and fragrance component [131]. It is the primary component of cinnamon and comprises over 98% of the essential oil found in cinnamon bark. A golden or amber thick liquid with a surprisingly pleasant scent is what is known as cinnamon essential oil [92]. Importantly, all naturally occurring cinnamaldehyde undergoes a structural transformation [93]. TRPA1 deletion increased mortality, cardiac dysfunction, and left ventricular remodelling in rats with DOX-induced dilated cardiomyopathy. A well-known TRPA1 activator, cinnamonaldehyde, improved cardiac function while lowering the inflammatory protein S100A8 production [94]. Mao *et al*. found that cinnamon aldehyde exhibited comparable cardioprotective effects in rats and DOX-treated H9c2 cells. By significantly raising HO-1 expression and encouraging Nrf2 nuclear translocation, cinnamon aldehyde reduced DOX-induced cardiomyocyte ferroptosis. However, the positive effects of cinnamon aldehyde on cardiomyocyte viability were counteracted by the presence of erastin, an agonist of ferroptosis [132].

### 4.14 Melatonin

All living things, including bacteria, plants, and animals, contain melatonin, a naturally occurring indoleamine [133]. The literature has extensively detailed the many functions of melatonin, including its ability to control circadian cycles. Moreover, it has anti-inflammatory, immunomodulatory, oncostatic, and vaso-regulatory properties [134]. Melatonin shields the heart against several cardiac conditions and has strong antioxidant effects on mitochondria. Melatonin showed promise as a treatment for DOX-induced cardiotoxicity in zebrafish, SD rats, and H9c2 cardiomyocyte models, according to Sun *et al*. By altering the yes-associated protein/Acyl-CoA synthetase long-chain family member 4 (YAP/ACSL4) axis, melatonin prevented DOX-induced cytotoxicity, apoptosis, mitochondrial malfunction, and ferroptosis [135]. Another research found that pretreatment with melatonin and the well-known iron chelator deferoxamine may reduce acute cardiac damage caused by DOX by blocking ferritinophagy and ferroptosis. In addition to maintaining heart function, this intervention lessened the negative effects of deferoxamine.

## 5 CHALLENGES AND FUTURE PROSPECTS

### 5.1 Challenges

#### 5.1.1 Pharmacokinetics and Bioavailability

Several phytochemicals are ineffective *in vivo* due to their low bioavailability. Some of the limitations include limited absorption, poor solubility, and quick metabolism, all of which reduce therapeutic potential. Although further research and clinical testing are required, techniques such as liposomal delivery, nanoformulations, and phytosome technology are being examined.

#### 5.1.2 Complexity of Mechanism

Ferroptosis is controlled by several processes, including oxidative stress, lipid peroxidation, and iron metabolism. Because phytochemicals often target a wide spectrum of cells, it might be difficult to pinpoint their mechanisms of action. Ferroptosis interacts with other cell death processes, including necroptosis and apoptosis, making interpretation more challenging. Advanced molecular biology technologies are necessary to offer a detailed understanding of these processes.

#### 5.1.3 Control of Quality and Standardisation

Consistency and repeatability are difficult to achieve owing to changes in phytochemical composition caused by differences in plant origin, extraction procedures, and environmental conditions. The development of phytochemicals as reliable therapeutic agents necessitates standardised extraction processes and quality assurance systems.

#### 5.1.4 Insufficient Clinical Support

Although promising pre-clinical research on phytochemicals targeting ferroptosis has been undertaken, there is a significant dearth of clinical evidence. To determine safety, effectiveness, and recommended dosage in individuals with cardiovascular disease (CVD), extensive, randomised, and controlled human trials are required. These studies still face challenges in acquiring financing, regulatory approval, and developing good study designs.

#### 5.1.5 Regulatory Obstacles

Phytochemicals have unique regulatory challenges in terms of classification, approval processes, and safety criteria as compared to conventional medicines. It remains challenging for scientists and companies to navigate these regulatory pathways for herbal items that target ferroptosis.

#### 5.1.6 Poor Bioavailability and Stability

Many phytoconstituents, such as curcumin and quercetin, suffer from poor solubility, rapid metabolism, and low systemic absorption, which reduces their potential for effective disease targeting. Extensive first-pass metabolism in the liver and intestines further limits their bioavailability, often necessitating the administration of high doses to achieve a therapeutic effect.

#### 5.1.7 Drug optimization and Standardization

Phytoconstituents lack a standardized dosing regimen, as their concentration can vary depending on the plant source, extraction method, and formulation. Some compounds, such as flavonoids, exhibit a biphasic dose-response effect, where low doses are beneficial, but high doses may induce pro-oxidant effects.

### 5.2 Prospects for the Future

#### 5.2.1 Development of Drug Delivery Systems

Improved drug delivery strategies, such as liposomes, nanoparticles, and biodegradable polymers, may enhance phytochemical targeting and bioavailability. These improvements may enhance treatment outcomes by allowing for the attainment of optimal phytoconstituent concentrations at ferroptotic action sites.

#### 5.2.2 Customised Medical Procedures

Phenotypic and genetic studies may help tailor phytochemical-based therapy to each patient's specific characteristics. By adjusting ferroptosis, personalised medicine may be able to improve the selection, dosage, and combination of phytoconstituents for improved CVD therapy.

#### 5.2.3 Exploring Complementary Combinations

Research into phytochemical combinations that target various ferroptosis components, such as antioxidants and iron chelators, might lead to more successful therapies. Research into synergistic effects in preclinical and clinical settings might result in the creation of innovative combinations that are more successful in treating cardiovascular disease.

#### 5.2.4 Applying Artificial Intelligence (AI) in Phytochemistry

Research AI and machine learning may accelerate the development of new phytochemicals by analyzing massive datasets, anticipating bioactivity, and identifying suitable candidates who might alter ferroptosis pathways. Computational modelling may help to hasten the development of more effective and personalised phytochemical therapies.

#### 5.2.5 Combining Traditional Therapies.

Studies investigating the use of phytochemicals in combination with standard CVD medicines may provide integrated therapies that minimise ferroptosis and give further cardioprotection. This additional technique may enhance treatment outcomes while decreasing adverse effects by lowering the dose of standard drugs.

#### 5.2.6 More Regulations and Public Support

Increased public support and possibly regulatory changes regarding the funding and licensing of medications based on phytoconstituents may come from the growing interest in natural therapies. Collaboration among academics, corporations, and regulatory authorities may hasten the development of phytochemicals that target ferroptosis for medicinal purposes.

## CONCLUSION

The potential of phytoconstituents as therapeutic agents for ferroptosis in cardiovascular disease (CVD) is quite promising. Because of their inherent antioxidant qualities, capacity to chelate iron, and capacity to inhibit lipid peroxidation, phytochemicals provide a viable strategy for halting the oxidative and iron-dependent damage brought on by ferroptosis in cardiac and vascular tissues. Curcumin, quercetin, resveratrol, and berberine are phytochemicals that have so far shown notable effectiveness; these compounds may reduce ferroptosis and enhance cardiovascular outcomes. Notwithstanding these encouraging results, issues remain, such as inadequate bioavailability, a range of plant sources, and limited clinical validity. Advancements in drug delivery techniques, such as phytosomes and nanoformulations, are needed to overcome these challenges. Before these substances may be utilized therapeutically for patients with CVD, further standardization, including clinical research, is needed to demonstrate their distinct effectiveness and safety features. Future studies should concentrate on customized strategies, complementary pairings, and adding phytoconstituents to current therapies. These natural compounds may prove to be a complementary and feasible alternative to conventional cardiovascular medications as more is learned about ferroptosis processes and phytochemical interactions. This might improve the prevention and treatment results for cardiovascular disease.

## Figures and Tables

**Fig. (1):**
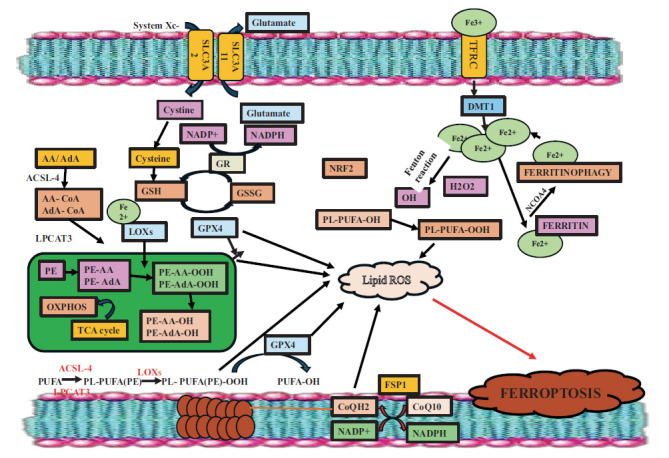
Molecular mechanisms governing ferroptosis. Lipid peroxidation, iron metabolism, and the GSH/GPX4 pathway are the three primary metabolic routes. The suppression of system Xc and depletion of GSH, or inhibition of GPX4, causes cell death and starts ferroptosis. The process of ferroptosis is controlled by lipid ROS. One crucial factor is found to be the peroxidation of PUFAs. Ferroptosis is caused by an excess of iron. Furthermore, the FSP1-CoQ10-NAD(P)H route, with its own mechanistic characteristics, participates in ferroptosis, according to recent studies. **Abbreviations:** AA, arachidonic acid; AdA, adrenoyl; ACSL4, Acyl-CoA synthetase long-chain family member 4; FSP1, ferroptosis suppressor protein 1; FPN1, ferroportin 1; GPX4, glutathione peroxidase 4; GSH, glutathione; DMT1, divalent metal transporter 1; LPCAT3, lysophosphatidylcholine acyltransferase 3; GSSG, oxidized glutathione; LOXs, lipoxygenases; GR, glutathione reductase; NCOA4, nuclear receptor coactivator 4; Tf, transferrin; PL, phospholipids.

**Fig. (2):**
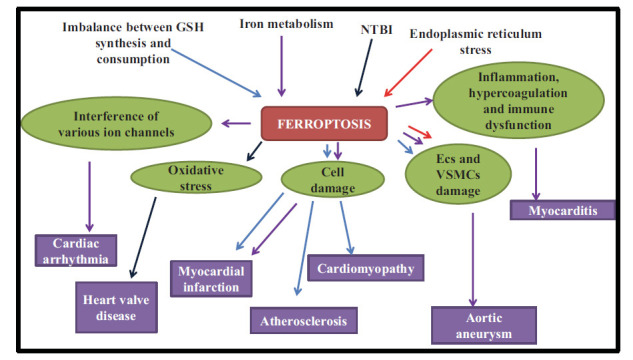
Ferroptosis as a key pathological mechanism contributing to cardiovascular disease.

**Table 1:** Herbals treatments target ferroptosis to treat CVD.

**Herb**	**Phytoconstituent**	**Mechanism of Action**	**Effect on** **Cardiovascular Disease**	**Type of CVD**	**References**
*Turmeric*	Curcumin	Reduces lipid peroxidation and ROS *via* the Naf2 pathway.	Reduces oxidative stress and cell death in cardiac cells.	Myocardial infarction, heart failure.	[55]
*Camellia sinensis*	Epigallocatechin gallate	Iron chelation and reducing lipid peroxidation.	Protects against endothelial dysfunction and atherosclerosis.	Atherosclerosis, hypertension.	[56]
*Glycyrrhiza glabra*	Glycyrrhizin	Inhibits lipid peroxidation and activates GPX4 enzyme.	Reduces inflammation and oxidative damage in myocardial cells.	Myocardial infaraction, cardiomyopathy.	[57]
*Scutellaria baicalenis*	Baicalin	Activates NRF2 pathway and enhances antioxidant defenses.	Preventing vascular damage and improving cardiac cell survival.	Vascular injury, atherosclerosis.	[58]
*Berberis vulgaris*	Berberine	Reduces lipid ROS and iron buildup to prevent ferroptosis.	Improves heart function and reduces ischemic disease.	Ischemic heart disease, myocardial infarction.	[59]
*Salvia miltiorrhiza*	Tanshinone IIA	Free radical scavengers reduce lipid peroxidation.	Protects myocardial tissue from oxidative injury.	Myocardial infarction, heart failure.	[60]
*Panax ginseng*	Ginsenosides	Iron chelation and inhibits ROS generation.	Reduces oxidative stress and myocardial apoptosis.	Herat failure, myocardial infarction.	[61]
*Ginkgo biloba*	Flavonoids and terpenoids	Inhibits lipid peroxidation and activates antioxidant pathways.	Enhances vascular function and reduces inflammation in heart tissue.	Peripheral artery disease, atherosclerosis	[62]
*Rosmarinus officinalis*	Rosmarinic acid, carnosic acid	Reduces lipid ROS levels and stabilizes cellular membrane.	Reduces cardiac inflammation and oxidative damage.	Myocardial infaraction, cardiomyopathy.	[63]
*Vitis vinifera*	Resveratrol	Activates Nrf2, reduces iron and induces lipid peroxidation	Protects against ischemic heart disease hyperfusion.	Ischemic heart disease.	[64]
*Allium sativum*	Allicin, diallyl sulfide	Scavenges ROS and regulates iron metabolism	Decreases lipid peroxidation and protects endothelial function.	Hypertension, atherosclerosis.	[65]
*Crataegus monogyna*	Flavonoids, proanthocyanidins	Inhibits lipid peroxidation and reduces ROS	Improves myocardial function and reduces ischemic injury.	Ischemic heart disease.	[66]
*Astragalus membranaceus*	Astragaloside IV	Activates Nrf2 pathway and reduces oxidative stress	Protects cardiac cells against apoptosis.	Heart failure, myocardial infarction.	[67]
*Rheum palmatum*	Emodin	Inhibits ferroptosis and reduces iron accumulation	Decreases cardiomyocyte injury during myocardial infarction.	Myocardial infarction	[68]
*Hypericum perforatum*	Hypericin, quercetin	Reduces lipid peroxidation and inhibits iron-mediated ROS	Reduces oxidative stress in endothelial cells.	Atherosclerosis.	[69]
*Cinnamomum cassia*	Cinnamaldehyde, eugenol	Reduces lipid peroxidation and activates Nrf2	Protects against oxidative stress and improves cardiac function.	Heart failure, atherosclerosis.	[70]
*Nigella sativa*	Thymoquinone	Inhibits lipid peroxidation and reduces ROS.	Prevents endothelial cell damage and reduces inflammation.	Atherosclerosis, myocardial ischemia	[71]

## LIST OF ABBREVIATIONS

7-KC: 7-ketocholesterol
ATF4: Activating Transcription Factor 4
CHOP: C/EBP Homologous Protein
CVD: Cardiovascular Disease
DOX: Doxorubicin
ECs: Endothelial Cells
EIF2α: Eukaryotic Initiation Factor 2 Alpha
ERS: Endoplasmic Reticulum Stress
FTH1: Ferritin Heavy Chain 1
GPX4: Glutathione Peroxidase 4
GSH: Glutathione
HO-1: Heme Oxygenase 1
Lip-ROS: Lipid Reactive Oxygen Species
LPC: Lysophosphatidylcholine
NRF2: Nuclear Factor Erythroid 2-related Factor 2
NTBI: Non-transferrin Bound Iron
PERK: Protein Kinase R-like Endoplasmic Reticulum Kinase
SLC7A11: Solute Carrier Family 7 Member 11 (also known as xCT)
TFR1: Transferrin Receptor 1
TRPA1: Transient Receptor Potential Ankyrin 1
VSMCs: Vascular Smooth Muscle Cells

## References

[1] Benjamin E.J., Blaha M.J., Chiuve S.E., Cushman M., Das S.R., Deo R. (2017). Heart disease and stroke statistics-2017 update: A report from the American heart association.. Circulation.

[2] Roth G.A., Mensah G.A., Johnson C.O. (2020). Global burden of cardiovascular diseases and risk factors, 1990-2019: Update from the GBD 2019 study.. J. Am. Coll. Cardiol..

[3] Dixon S.J., Lemberg K.M., Lamprecht M.R., Skouta R., Zaitsev E.M., Gleason C.E. (2012). Ferroptosis: An iron-dependent form of nonapoptotic cell death.. Cell.

[4] Winterbourn C.C. (1995). Toxicity of iron and hydrogen peroxide: The Fenton reaction.. Toxicol. Lett..

[5] Stockwell B.R., Friedmann Angeli J.P., Bayir H. (2017). Ferroptosis: A regulated cell death nexus linking metabolism, redox biology, and disease.. Cell.

[6] Kagan V.E., Mao G., Qu F. (2017). Oxidized arachidonic and adrenic PEs navigate cells to ferroptosis.. Nat. Chem. Biol..

[7] Esterbauer H., Zollern H. (1989). Methods for determination of aldehydic lipid peroxidation products.. Free Radic. Biol. Med..

[8] Zhang H.L., Hu B.X., Li Z.L. (2022). PKCβII phosphorylates ACSL4 to amplify lipid peroxidation to induce ferroptosis.. Nat. Cell Biol..

[9] Doll S., Proneth B., Tyurina Y.Y. (2017). ACSL4 dictates ferroptosis sensitivity by shaping cellular lipid composition.. Nat. Chem. Biol..

[10] Yang W.S., Kim K.J., Gaschler M.M., Patel M., Shchepinov M.S., Stockwell B.R. (2016). Peroxidation of polyunsaturated fatty acids by lipoxygenases drives ferroptosis.. Proc. Natl. Acad. Sci. USA.

[11] McBean G.J. (2012). The transsulfuration pathway: A source of cysteine for glutathione in astrocytes.. Amino Acids.

[12] Shimada K., Stockwell B.R. (2016). tRNA synthase suppression activates de novo cysteine synthesis to compensate for cystine and glutathione deprivation during ferroptosis.. Mol. Cell. Oncol..

[13] Hayano M., Yang W.S., Corn C.K., Pagano N.C., Stockwell B.R. (2016). Loss of cysteinyl-tRNA synthetase (CARS) induces the transsulfuration pathway and inhibits ferroptosis induced by cystine deprivation.. Cell Death Differ..

[14] Gao M., Monian P., Quadri N., Ramasamy R., Jiang X. (2015). Glutaminolysis and transferrin regulate ferroptosis.. Mol. Cell.

[15] Zhao Y., Hu X., Liu Y. (2017). ROS signaling under metabolic stress: Cross-talk between AMPK and AKT pathway.. Mol. Cancer.

[16] Sun X., Ou Z., Chen R. (2016). Activation of the p62‐Keap1‐NRF2 pathway protects against ferroptosis in hepatocellular carcinoma cells.. Hepatology.

[17] Bedard K., Krause K.H. (2007). The NOX family of ROS-generating NADPH oxidases: Physiology and pathophysiology.. Physiol. Rev..

[18] Magtanong L., Ko P.J., Dixon S.J. (2016). Emerging roles for lipids in non-apoptotic cell death.. Cell Death Differ..

[19] Fan Z., Wirth A-K., Chen D. (2017). Nrf2-Keap1 pathway promotes cell proliferation and diminishes ferroptosis.. Oncogenesis.

[20] Friedmann Angeli J.P., Schneider M., Proneth B. (2014). Inactivation of the ferroptosis regulator Gpx4 triggers acute renal failure in mice.. Nat. Cell Biol..

[21] Chen H., Attieh Z.K., Su T. (2004). Hephaestin is a ferroxidase that maintains partial activity in sex-linked anemia mice.. Blood.

[22] Levy J.E., Jin O., Fujiwara Y., Kuo F., Andrews N. (1999). Transferrin receptor is necessary for development of erythrocytes and the nervous system.. Nat. Genet..

[23] Stoyanovsky D.A., Tyurina Y.Y., Shrivastava I. (2019). Iron catalysis of lipid peroxidation in ferroptosis: Regulated enzymatic or random free radical reaction?. Free Radic. Biol. Med..

[24] Liochev S.I. (1999). The mechanism of “Fenton-like” reactions and their importance for biological systems. A biologist’s view.. Met. Ions Biol. Syst..

[25] Yang W.S., Stockwell B.R. (2008). Synthetic lethal screening identifies compounds activating iron-dependent, nonapoptotic cell death in oncogenic-RAS-harboring cancer cells.. Chem. Biol..

[26] Gao M., Monian P., Jiang X. (2015). Metabolism and iron signaling in ferroptotic cell death.. Oncotarget.

[27] Hirota K. (2019). An intimate crosstalk between iron homeostasis and oxygen metabolism regulated by the hypoxia-inducible factors (HIFs).. Free Radic. Biol. Med..

[28] Shindou H., Shimizu T. (2009). Acyl-CoA:lysophospholipid acyltransferases.. J. Biol. Chem..

[29] Gammella E., Recalcati S., Rybinska I., Buratti P., Cairo G. (2015). Iron-induced damage in cardiomyopathy: Oxidative-dependent and independent mechanisms.. Oxid. Med. Cell. Longev..

[30] Hou W., Xie Y., Song X. (2016). Autophagy promotes ferroptosis by degradation of ferritin.. Autophagy.

[31] Silva A.M.N., Rangel M. (2022). The (bio)chemistry of non-transferrin-bound iron.. Molecules.

[32] Porter J.B., Walter P.B., Neumayr L.D. (2014). Mechanisms of plasma non‐transferrin bound iron generation: Insights from comparing transfused diamond blackfan anaemia with sickle cell and thalassaemia patients.. Br. J. Haematol..

[33] Loréal O., Gosriwatana I., Guyader D., Porter J., Brissot P., Hider R.C. (2000). Determination of non-transferrin-bound iron in genetic hemochromatosis using a new HPLC-based method.. J. Hepatol..

[34] Aruoma O.I., Bomford A., Polson R.J., Halliwell B. (1988). Nontransferrin-bound iron in plasma from hemochromatosis patients: Effect of phlebotomy therapy.. Blood.

[35] Van Campenhout A., Van Campenhout C., Lagrou A.R., Moorkens G., De Block C., Manuel-y-Keenoy B. (2006). Iron-binding antioxidant capacity is impaired in diabetes mellitus.. Free Radic. Biol. Med..

[36] Hershko C., Graham G., Bates G.W., Rachmilewitz E.A. (1978). Non-specific serum iron in thalassaemia: an abnormal serum iron fraction of potential toxicity.. Br. J. Haematol..

[37] Jiang L.R., Shen M.Q., Bao Y.X., Qian Z.M. (2022). Verapamil downregulates iron uptake and upregulates divalent metal transporter 1 expression in H9C2 cardiomyocytes.. Fundam. Clin. Pharmacol..

[38] Kumfu S., Chattipakorn S., Srichairatanakool S., Settakorn J., Fucharoen S., Chattipakorn N. (2011). T-type calcium channel as a portal of iron uptake into cardiomyocytes of beta-thalassemic mice.. Eur. J. Haematol..

[39] Kumfu S., Chattipakorn S., Fucharoen S., Chattipakorn N. (2013). Ferric iron uptake into cardiomyocytes of β-thalassemic mice is not through calcium channels.. Drug Chem. Toxicol..

[40] Nawi A.M., Chin S.F., Azhar Shah S., Jamal R. (2019). Tissue and serum trace elements concentration among colorectal patients: A systematic review of case-control studies.. Iran. J. Public Health.

[41] Liu G., Xie X., Liao W. (2024). Ferroptosis in cardiovascular disease.. Biomed. Pharmacother..

[42] Wang Y., Kuang X., Yin Y. (2022). Tongxinluo prevents chronic obstructive pulmonary disease complicated with atherosclerosis by inhibiting ferroptosis and protecting against pulmonary microvascular barrier dysfunction.. Biomed. Pharmacother..

[43] Wang X., Zhang M., Mao C. (2023). Icariin alleviates ferroptosis‐related atherosclerosis by promoting autophagy in xo‐LDL‐induced vascular endothelial cell injury and atherosclerotic mice.. Phytother. Res..

[44] Rong J., Li C., Zhang Q. (2023). Hydroxysafflor yellow A inhibits endothelial cell ferroptosis in diabetic atherosclerosis mice by regulating miR-429/SLC7A11.. Pharm. Biol..

[45] Mylonas K.S., Peroulis M., Schizas D., Kapelouzou A. (2023). MYD88 and proinflammatory chemokines in aortic atheromatosis: Exploring novel statin effects.. Int. J. Mol. Sci..

[46] Li Q., Liu C., Deng L. (2021). Novel function of fluvastatin in attenuating oxidized low density lipoprotein induced endothelial cell ferroptosis in a glutathione peroxidase4 and cystine glutamate antiporter dependent manner.. Exp. Ther. Med..

[47] Fang X., Wang H., Han D. (2019). Ferroptosis as a target for protection against cardiomyopathy.. Proc. Natl. Acad. Sci. USA.

[48] Park T.J., Park J.H., Lee G.S. (2019). Quantitative proteomic analyses reveal that GPX4 downregulation during myocardial infarction contributes to ferroptosis in cardiomyocytes.. Cell Death Dis..

[49] Gorrini C., Harris I.S., Mak T.W. (2013). Modulation of oxidative stress as an anticancer strategy.. Nat. Rev. Drug Discov..

[50] Dodson M., Castro-Portuguez R., Zhang D.D. (2019). NRF2 plays a critical role in mitigating lipid peroxidation and ferroptosis.. Redox Biol..

[51] Viswanathan V.S., Ryan M.J., Dhruv H.D. (2017). Dependency of a therapy-resistant state of cancer cells on a lipid peroxidase pathway.. Nature.

[52] Bulluck H., Rosmini S., Abdel-Gadir A. (2016). Residual myocardial iron following intramyocardial hemorrhage during the convalescent phase of reperfused ST-segment-elevation myocardial infarction and adverse left ventricular remodeling.. Circ. Cardiovasc. Imaging.

[53] Chen X., Xu S., Zhao C., Liu B. (2019). Role of TLR4/NADPH oxidase 4 pathway in promoting cell death through autophagy and ferroptosis during heart failure.. Biochem. Biophys. Res. Commun..

[54] Liu B., Zhao C., Li H., Chen X., Ding Y., Xu S. (2018). Puerarin protects against heart failure induced by pressure overload through mitigation of ferroptosis.. Biochem. Biophys. Res. Commun..

[55] Zhou N., Wei S., Sun T. (2023). Recent progress in the role of endogenous metal ions in doxorubicin-induced cardiotoxicity.. Front. Pharmacol..

[56] Oyama J., Shiraki A., Nishikido T. (2017). EGCG, a green tea catechin, attenuates the progression of heart failure induced by the heart/muscle-specific deletion of MnSOD in mice.. J. Cardiol..

[57] Zhu K., Fan R., Cao Y. (2024). Glycyrrhizin attenuates myocardial ischemia reperfusion injury by suppressing Inflammation, oxidative stress, and ferroptosis via the HMGB1-TLR4-GPX4 pathway.. Exp. Cell Res..

[58] Fan Z., Cai L., Wang S., Wang J., Chen B. (2021). Baicalin prevents myocardial ischemia/reperfusion injury through inhibiting ACSL4 mediated ferroptosis.. Front. Pharmacol..

[59] Yang K.T., Chao T.H., Wang I.C. (2022). Berberine protects cardiac cells against ferroptosis.. Tzu-Chi Med. J..

[60] Hu T., Zou H.X., Le S.Y. (2023). Tanshinone IIA confers protection against myocardial ischemia/reperfusion injury by inhibiting ferroptosis and apoptosis via VDAC1.. Int. J. Mol. Med..

[61] Chen J., Huang Q., Li J. (2023). Panax ginseng against myocardial ischemia/reperfusion injury: A review of preclinical evidence and potential mechanisms.. J. Ethnopharmacol..

[62] Ye W., Wang J., Little P.J. (2024). Anti-atherosclerotic effects and molecular targets of ginkgolide B from Ginkgo biloba.. Acta Pharm. Sin. B.

[63] Wang B., Wang J., Liu C., Li C., Meng T., Chen J. (2024). Ferroptosis: Latest evidence and perspectives on plant‐derived natural active compounds mitigating doxorubicin‐induced cardiotoxicity.. J. Appl. Toxicol..

[64] Li T., Tan Y., Ouyang S., He J., Liu L. (2022). Resveratrol protects against myocardial ischemia-reperfusion injury via attenuating ferroptosis.. Gene.

[65] Wang K., Dong Y., Liu J. (2020). Effects of REDOX in regulating and treatment of metabolic and inflammatory cardiovascular diseases.. Oxid. Med. Cell. Longev..

[66] Huang P., Wan H., Shao C., Li C., Zhang L., He Y. (2022). Recent advances in Chinese herbal medicine for cerebral ischemic reperfusion injury.. Front. Pharmacol..

[67] Jiang M., Ni J., Cao Y., Xing X., Wu Q., Fan G. (2019). Astragaloside IV attenuates myocardial ischemia‐reperfusion injury from oxidative stress by regulating succinate, lysophospholipid metabolism, and ROS scavenging system.. Oxid. Med. Cell. Longev..

[68] Hu S., Zhou J., Hao J. (2024). Emodin ameliorates doxorubicin-induced cardiotoxicity by inhibiting ferroptosis through the remodeling of gut microbiota composition.. Am. J. Physiol. Cell Physiol..

[69] Bellavite P., Fazio S., Affuso F. (2023). A descriptive review of the action mechanisms of berberine, quercetin and silymarin on insulin resistance/hyperinsulinemia and cardiovascular prevention.. Molecules.

[70] Lu L., Xiong Y., Zhou J., Wang G., Mi B., Liu G. (2022). The therapeutic roles of cinnamaldehyde against cardiovascular diseases.. Oxid. Med. Cell. Longev..

[71] Zhang T., Deng W., Deng Y. (2023). Mechanisms of ferroptosis regulating oxidative stress and energy metabolism in myocardial ischemia-reperfusion injury and a novel perspective of natural plant active ingredients for its treatment.. Biomed. Pharmacother..

[72] Bonnefont-Rousselot D. (2016). Resveratrol and cardiovascular diseases.. Nutrients.

[73] Malaguarnera L. (2019). Influence of resveratrol on the immune response.. Nutrients.

[74] Hu L.F., Lan H.R., Li X.M., Jin K.T. (2021). A systematic review of the potential chemoprotective effects of resveratrol on doxorubicin-induced cardiotoxicity: Focus on the antioxidant, antiapoptotic, and anti-inflammatory activities.. Oxid. Med. Cell. Longev..

[75] Yu W., Chen C., Xu C. (2022). Activation of p62-NRF2 axis protects against doxorubicininduced ferroptosis in cardiomyocytes: A novel role and molecular mechanism of resveratrol.. Am. J. Chin. Med..

[76] Chen L., Sun X., Wang Z. (2024). Resveratrol protects against doxorubicin-induced cardiotoxicity by attenuating ferroptosis through modulating the MAPK signaling pathway.. Toxicol. Appl. Pharmacol..

[77] Li Y., Meeran S.M., Tollefsbol T.O. (2017). Combinatorial bioactive botanicals re-sensitize tamoxifen treatment in ER-negative breast cancer via epigenetic reactivation of ERα expression.. Sci. Rep..

[78] Wang L., Li P., Feng K. (2023). EGCG adjuvant chemotherapy: Current status and future perspectives.. Eur. J. Med. Chem..

[79] Li W., Nie S., Xie M., Chen Y., Li C., Zhang H. (2010). A major green tea component, (-)-epigallocatechin-3-gallate, ameliorates doxorubicin-mediated cardiotoxicity in cardiomyocytes of neonatal rats.. J. Agric. Food Chem..

[80] Zheng J., Lee H.C., Bin Sattar M.M., Huang Y., Bian J.S. (2011). Cardioprotective effects of epigallocatechin-3-gallate against doxorubicin-induced cardiomyocyte injury.. Eur. J. Pharmacol..

[81] Saeed N.M., El-Naga R.N., El-Bakly W.M., Abdel-Rahman H.M., Salah ElDin R.A., El-Demerdash E. (2015). Epigallocatechin-3-gallate pretreatment attenuates doxorubicin-induced cardiotoxicity in rats: A mechanistic study.. Biochem. Pharmacol..

[82] Yao Y.F., Liu X., Li W.J. (2017). (−)-Epigallocatechin-3-gallate alleviates doxorubicin-induced cardiotoxicity in sarcoma 180 tumor-bearing mice.. Life Sci..

[83] He H., Wang L., Qiao Y., Yang B., Yin D., He M. (2021). Epigallocatechin-3-gallate pretreatment alleviates doxorubicin-induced ferroptosis and cardiotoxicity by upregulating AMPKα2 and activating adaptive autophagy.. Redox Biol..

[84] Kwiecień I., Miceli N., D’Arrigo M., Marino A., Ekiert H. (2022). Antioxidant potential and enhancement of bioactive metabolite production in in vitro cultures of Scutellaria lateriflora L. by biotechnological methods.. Molecules.

[85] Hu Q., Zhang W., Wu Z. (2021). Baicalin and the liver-gut system: Pharmacological bases explaining its therapeutic effects.. Pharmacol. Res..

[86] El-Ela S.R.A., Zaghloul R.A., Eissa L.A. (2022). Promising cardioprotective effect of baicalin in doxorubicin-induced cardiotoxicity through targeting toll-like receptor 4/nuclear factor-κB and Wnt/β-catenin pathways.. Nutrition.

[87] Feng P., Yang Y., Liu N., Wang S. (2022). Baicalin regulates TLR4/IκBα/NFκB signaling pathway to alleviate inflammation in Doxorubicin related cardiotoxicity.. Biochem. Biophys. Res. Commun..

[88] Zeng Y., Liao X., Guo Y. (2024). Baicalin-peptide supramolecular self-assembled nanofibers effectively inhibit ferroptosis and attenuate doxorubicin-induced cardiotoxicity.. J. Control. Release.

[89] Wongnen C., Ruzzama N., Chaijan M., Cheong L.Z., Panpipat W. (2022). Glochidion wallichianum leaf extract as a natural antioxidant in sausage model system.. Foods.

[90] Zhu R., Liu H., Liu C. (2017). Cinnamaldehyde in diabetes: A review of pharmacology, pharmacokinetics and safety.. Pharmacol. Res..

[91] Li L., Zhang W., Peng J. (2020). A novel shell material-highland barley starch for microencapsulation of cinnamon essential oiwith different preparation methods.. Materials.

[92] Nile A., Shin J., Shin J. (2023). Cinnamaldehyde-rich cinnamon extract induces cell death in colon cancer cell lines HCT 116 and HT-29.. Int. J. Mol. Sci..

[93] Zhao M., Zheng Z., Pan W. (2023). TRPA1 deficiency aggravates dilated cardiomyopathy by promoting S100A8 expression to induce M1 macrophage polarization in rats.. FASEB J..

[94] Mao M., Zheng W., Deng B. (2023). Cinnamaldehyde alleviates doxorubicin-induced cardiotoxicity by decreasing oxidative stress and ferroptosis in cardiomyocytes.. PLoS One.

[95] Musolino M., D’Agostino M., Zicarelli M., Andreucci M., Coppolino G., Bolignano D. (2024). Spice up your kidney: A review on the effects of capsaicin in renal physiology and disease.. Int. J. Mol. Sci..

[96] López M., Quintero-Macías L., Huerta M. (2022). Capsaicin decreases kidney iron deposits and increases hepcidin levels in diabetic rats with iron overload: A preliminary study.. Molecules.

[97] Wang L., Liu Y., Li S. (2023). Capsaicin alleviates doxorubicin-induced acute myocardial injury by regulating iron homeostasis and PI3K-Akt signaling pathway.. Aging.

[98] Jia S., Xu X., Zhou S., Chen Y., Ding G., Cao L. (2019). Fisetin induces autophagy in pancreatic cancer cells via endoplasmic reticulum stress- and mitochondrial stress-dependent pathways.. Cell Death Dis..

[99] Kumar R.M., Kumar H., Bhatt T. (2023). Fisetin in cancer: attributes, developmental aspects, and nanotherapeutics.. Pharmaceuticals.

[100] Ma T., Kandhare A.D., Mukherjee-Kandhare A.A., Bodhankar S.L. (2019). Fisetin, a plant flavonoid ameliorates doxorubicin-induced cardiotoxicity in experimental rats: The decisive role of caspase-3, COX-II, cTn-I, iNOs and TNF-α.. Mol. Biol. Rep..

[101] Lin K.H., Ramesh S., Agarwal S. (2023). Fisetin attenuates doxorubicin‐induced cardiotoxicity by inhibiting the insulin‐like growth factor II receptor apoptotic pathway through estrogen receptor‐α/‐β activation.. Phytother. Res..

[102] Li D., Liu X., Pi W. (2022). Fisetin attenuates doxorubicin-induced cardiomyopathy in vivo and in vitro by inhibiting ferroptosis through SIRT1/Nrf2 signaling pathway activation.. Front. Pharmacol..

[103] Chen X., Liu Z., Meng R., Shi C., Guo N. (2017). Antioxidative and anticancer properties of Licochalcone A from licorice.. J. Ethnopharmacol..

[104] Li M.T., Xie L., Jiang H.M. (2022). Role of licochalcone A in potential pharmacological therapy: A review.. Front. Pharmacol..

[105] de Freitas K.S., Squarisi I.S., Acésio N.O. (2020). Licochalcone A, a licorice flavonoid: Antioxidant, cytotoxic, genotoxic, and chemopreventive potential.. J. Toxicol. Environ. Health A.

[106] Chen G., Luo S., Guo H., Lin J., Xu S. (2023). Licochalcone A alleviates ferroptosis in doxorubicin-induced cardiotoxicity via the PI3K/AKT/MDM2/p53 pathway.. Naunyn Schmiedebergs Arch. Pharmacol..

[107] Thuan N.H., Shrestha A., Trung N.T. (2022). Advances in biochemistry and the biotechnological production of taxifolin and its derivatives.. Biotechnol. Appl. Biochem..

[108] Liu Y., Shi X., Tian Y. (2023). An insight into novel therapeutic potentials of taxifolin.. Front. Pharmacol..

[109] Alzaharna M., Alqouqa I., Cheung H.Y. (2017). Taxifolin synergizes Andrographolide-induced cell death by attenuation of autophagy and augmentation of caspase dependent and independent cell death in HeLa cells.. PLoS One.

[110] Unver E., Tosun M., Olmez H., Kuzucu M., Cimen F.K., Suleyman Z. (2019). The effect of taxifolin on cisplatin-induced pulmonary damage in rats: A biochemical and histopathological evaluation.. Mediators Inflamm..

[111] Lin Z., Wang J. (2023). Taxifolin protects against doxorubicin-induced cardiotoxicity and ferroptosis by adjusting microRNA-200a-mediated Nrf2 signaling pathway.. Heliyon.

[112] Liu Y., Tang B.L., Lu M.L., Wang H.X. (2023). Astragaloside IV improves pulmonary arterial hypertension by increasing the expression of CCN1 and activating the ERK1/2 pathway.. J. Cell. Mol. Med..

[113] Zhang Y., Zhang Y., Jin X. (2019). The role of Astragaloside IV against cerebral ischemia/reperfusion injury: Suppression of apoptosis via promotion of P62-LC3-autophagy.. Molecules.

[114] Jia Y., Zuo D., Li Z. (2014). Astragaloside IV inhibits doxorubicin-induced cardiomyocyte apoptosis mediated by mitochondrial apoptotic pathway via activating the PI3K/Akt pathway.. Chem. Pharm. Bull..

[115] Lin J., Fang L., Li H. (2019). Astragaloside IV alleviates doxorubicin induced cardiomyopathy by inhibiting NADPH oxidase derived oxidative stress.. Eur. J. Pharmacol..

[116] Chen X., Tian C., Zhang Z. (2023). Astragaloside IV inhibits NLRP3 inflammasome-mediated pyroptosis via activation of Nrf-2/HO-1 signaling pathway and protects against doxorubicin-induced cardiac dysfunction.. Front. Biosci..

[117] Luo L.F., Guan P., Qin L.Y., Wang J.X., Wang N. (2021). Ji ES. Astragaloside IV inhibits adriamycin-induced cardiac ferroptosis by enhancing Nrf2 signaling.. Mol. Cell. Biochem..

[118] Gackowski M., Szewczyk-Golec K., Mądra-Gackowska K., Pluskota R., Koba M. (2022). Quantitative structure-activity relationship analysis of isosteviol-related compounds as activated coagulation factor X (FXa) inhibitors.. Nutrients.

[119] Wang M., Li H., Xu F. (2018). Diterpenoid lead stevioside and its hydrolysis products steviol and isosteviol: Biological activity and structural modification.. Eur. J. Med. Chem..

[120] Xu C., Ou E., Li Z. (2022). Synthesis and in vivo evaluation of new steviol derivatives that protect against cardiomyopathy by inhibiting ferroptosis.. Bioorg. Chem..

[121] Dong X., Fu J., Yin X. (2016). Emodin: A review of its pharmacology, toxicity and pharmacokinetics.. Phytother. Res..

[122] HaoShang (2023). HaoShang, Jia X, Liu H, Zhang X, Shao Y. A comprehensive review of emodin in fibrosis treatment.. Fitoterapia.

[123] Dai S., Chen Y., Fan X. (2023). Emodin attenuates cardiomyocyte pyroptosis in doxorubicin-induced cardiotoxicity by directly binding to GSDMD.. Phytomedicine.

[124] Xian M., Cai J., Zheng K. (2021). Aloe-emodin prevents nerve injury and neuroinflammation caused by ischemic stroke via the PI3K/AKT/mTOR and NF-κB pathway.. Food Funct..

[125] Dong X., Zeng Y., Liu Y. (2020). Aloe‐emodin: A review of its pharmacology, toxicity, and pharmacokinetics.. Phytother. Res..

[126] Wu M., Ling W., Wei J. (2022). Biomimetic photosensitizer nanocrystals trigger enhanced ferroptosis for improving cancer treatment.. J. Control. Release.

[127] He Y., Xi J., Fang J., Zhang B., Cai W. (2023). Aloe-emodin alleviates doxorubicin-induced cardiotoxicity via inhibition of ferroptosis.. Free Radic. Biol. Med..

[128] Li J., Yu H., Yang C., Ma T., Dai Y. (2022). Therapeutic potential and molecular mechanisms of echinacoside in neurodegenerative diseases.. Front. Pharmacol..

[129] Wang W., Jiang S., Zhao Y., Zhu G. (2023). Echinacoside: A promising active natural products and pharmacological agents.. Pharmacol. Res..

[130] Ma Y., Yang X., Jiang N., Lu C., Zhang J., Zhuang S. (2023). Echinacoside ameliorates doxorubicin induced cardiac injury by regulating GPX4 inhibition induced ferroptosis.. Exp. Ther. Med..

[131] Li L., Zhang W., Peng J. (2020). A novel shell material-highland barley starch for microencapsulation of cinnamon essential oil with different preparation methods.. Materials.

[132] Morcillo-Parra M.Á., Beltran G., Mas A., Torija M.J. (2019). Determination of melatonin by a whole cell bioassay in fermented beverages.. Sci. Rep..

[133] Talib W.H. (2018). Melatonin and cancer hallmarks.. Molecules.

[134] Sun X., Sun P., Zhen D. (2022). Melatonin alleviates doxorubicin-induced mitochondrial oxidative damage and ferroptosis in cardiomyocytes by regulating YAP expression.. Toxicol. Appl. Pharmacol..

[135] Hanna M., Seddiek H., Aboulhoda B.E. (2022). Synergistic cardioprotective effects of melatonin and deferoxamine through the improvement of ferritinophagy in doxorubicin-induced acute cardiotoxicity.. Front. Physiol..

